# Coordination among the rearfoot, midfoot, and forefoot during walking

**DOI:** 10.1186/s13047-017-0224-3

**Published:** 2017-09-25

**Authors:** Tomoya Takabayashi, Mutsuaki Edama, Emi Nakamura, Erika Yokoyama, Chiaki Kanaya, Masayoshi Kubo

**Affiliations:** Institute for Human Movement and Medical Sciences, Niigata University of Health and Welfare, 1398 Shimami-cho, Kita-Ku, Niigata City, Niigata, 950-3198 Japan

**Keywords:** Modified vector coding technique, Coordination pattern classification, Midfoot, Walking

## Abstract

**Background:**

Examining coordination between segments is essential for prevention and treatment of injuries. However, traditional methods such as ratio, cross-correlation technique, and angle-time plot may not provide a complete understanding of intersegmental coordination. The present study aimed to quantify the coordination among the rearfoot, midfoot, and forefoot segments during walking.

**Methods:**

Twenty healthy young men walked barefoot on a treadmill. Reflective markers were fixed to their right shank and foot based on the Leardini foot model. Three-dimensional joint angles were calculated at the distal segment, and were expressed relative to the adjacent proximal segment. The coupling angle representing intersegmental coordination was calculated by using the modified vector coding technique, and categorized into the following four coordination patterns: in-phase with proximal dominancy, in-phase with distal dominancy, tanti-phase with proximal dominancy, and anti-phase with distal dominancy.

**Results:**

The results showed that the midfoot was dominantly everted compared with the rearfoot and forefoot during the early stance (i.e., the rearfoot-midfoot coordination and midfoot-forefoot coordination were mainly in-phase with distal and proximal dominancy, respectively).

**Conclusion:**

This result may suggest that the midfoot plays a more significant role than the rearfoot and forefoot during early stance. The results of the present study can help in understanding the interaction of the intersegmental foot kinematic time series during walking. The results could be used as data to distinguish the presence of injuries or abnormal inter-segmental foot motions such as pes planus. Additionally, these data might be used in the future in a comparison with data on foot deformities.

## Background

Pathologies of foot structures, such as pes planus, pes cavus, and first metatarsal-phalangeal joint stiffness, affect foot kinematics at the site of the impaired structure during gait [1, 2]. Additionally, such localized abnormal motion of one segment may also influence kinematics in the other segment. Such information on kinematic coupling between segments is often quantified using the Pearson correlation-coefficient [3], or cross-correlation coefficients [4]. However, the limitation of these techniques is that they only provide an indication of temporal similarity between two angular waveforms throughout the stance phase [5]. For instance, while two angular waveforms undergoing similar directional movement patterns will produce a high correlation coefficient, they might have very different angular amplitudes. Hence, correlation methods cannot adequately evaluate angular amplitude parameters, such as peak values and range of motion.

Many studies [6–8] have used the ratio of angular peak values in two segments to investigate kinematic coupling. Nawoczenski et al. [7] found that runners with pes cavus with an associated lower ratio between the peak rearfoot eversion angle and peak tibia internal rotation (i.e., relatively small rearfoot motion) showed a greater incidence of foot injuries. However, although this method uses the range of motion calculated from the value at initial contact to the peak angular value, the timings of peak angular value are inherently different in the two segments [9]. Additionally, this method cannot be used for comparing time series (e.g., early stance vs. midstance) because the ratio assigns a single value for the whole stance [10].

One of the methods available to investigate kinematic coupling that can overcome these limitations is the vector coding technique [11]. This technique is capable of quantifying the angular amplitude dominancy in the proximal or distal segment continuously during the stance phase [12, 13]. Chang et al. [13] expanded this technique and classified the coordination patterns between the rearfoot and forefoot during walking as anti-phase (adjoining segments rotate in the opposite direction), in-phase (adjoining segments rotate in the same direction), proximal-phase (rearfoot rotates dominancy), and distal-phase (forefoot rotates dominancy). Their study demonstrated that the push off during walking combined with the in-phase and anti-phase coordination in the frontal plane. In contrast, other studies [14, 15] have reported features of the stable foot during the push off as having an anti-phase pattern in rearfoot-forefoot coordination (rearfoot inversion and forefoot eversion). However, since this research does not use a modified vector coding technique, Chang et al. [13] pointed out that it oversimplifies the complexity of rearfoot and forefoot interactions.

Notably, in the classification method proposed in the study by Chang et al. [13], the dominancy of the angular amplitude in the segment cannot also be determined in all coordination patterns. For example, the coordination between the rearfoot and forefoot in the sagittal plane during the late stance is all in-phase (both segments with plantarflexion) [13]. However, this coordination pattern shows only the movement direction and does not provide the information about which segment is the dominant angular amplitude. While Arnold et al. [16] have categorized the coordination among the rearfoot, midfoot, and forefoot during walking between younger and older adults by using Chang’s classification method [13], this study provides no information on which segment is dominant. Recently, Needham et al. [17] offered a new coordination pattern, which contained the in-phase or anti-phase along with information on angular amplitude dominancy of segments.

Therefore, the purpose of the present study was to quantify the coordination of intersegmental foot kinematics during walking by using the modified vector coding technique and the new classification method of Needham et al. [18]. Dubbeldam et al. [3] investigated the kinematic coupling between the hallux and rearfoot while walking. However, because they did not include midfoot and forefoot motion, the mechanism with which the kinematic coupling between hallux and rearfoot occurs has not been known. Also, a previous study [16] investigated the coordination between the adjacent rearfoot and midfoot and between the adjacent midfoot and forefoot. Their rotation patterns were analyzed in the same plane, and other studies [13, 17] have performed the same analysis. Furthermore, we have shown that kinematic coupling between the adjacent rearfoot and midfoot and between the adjacent midfoot and forefoot in the frontal plane during walking have stronger coupling (*r* = 0.84, *r* = 0.75, respectively) than that of the opposing plane [19]. Considering these previous studies, the present study investigated coordination between the rearfoot and midfoot and between the midfoot and forefoot in the same planes. The results of the present study could help in improving the understanding the interaction among intersegmental foot kinematic time series, and in better facilitating the interpretation of coordination patterns.

## Methods

### Participants

Twenty healthy male participants (height 171.8 ± 5.1 cm; body mass 65.2 ± 7.8 kg; age 21.5 ± 2.5 years), without pes planus or other pathologies volunteered to participate in this study. Forefoot abduction is associated with flattening of the medial longitudinal arch [20], and the pes planus presents as an abduction of the forefoot during standing. Thus, the foot posture was evaluated using the “too many toes” sign [21], and participants who had been observed with one or more toes along the lateral aspect from the back in the standing position (i.e. forefoot abduction) were excluded. Based on this evaluation, as we excluded participants with pes planus, the target participants had only normal foot posture. Participants were recruited from the student population of Niigata University of Health and Welfare. All participants provided informed consent prior to participation. The present study was reviewed and approved by the ethical committee (No. 17575-150,422) at our institution.

### Experimental protocol

**Fig. 1:**
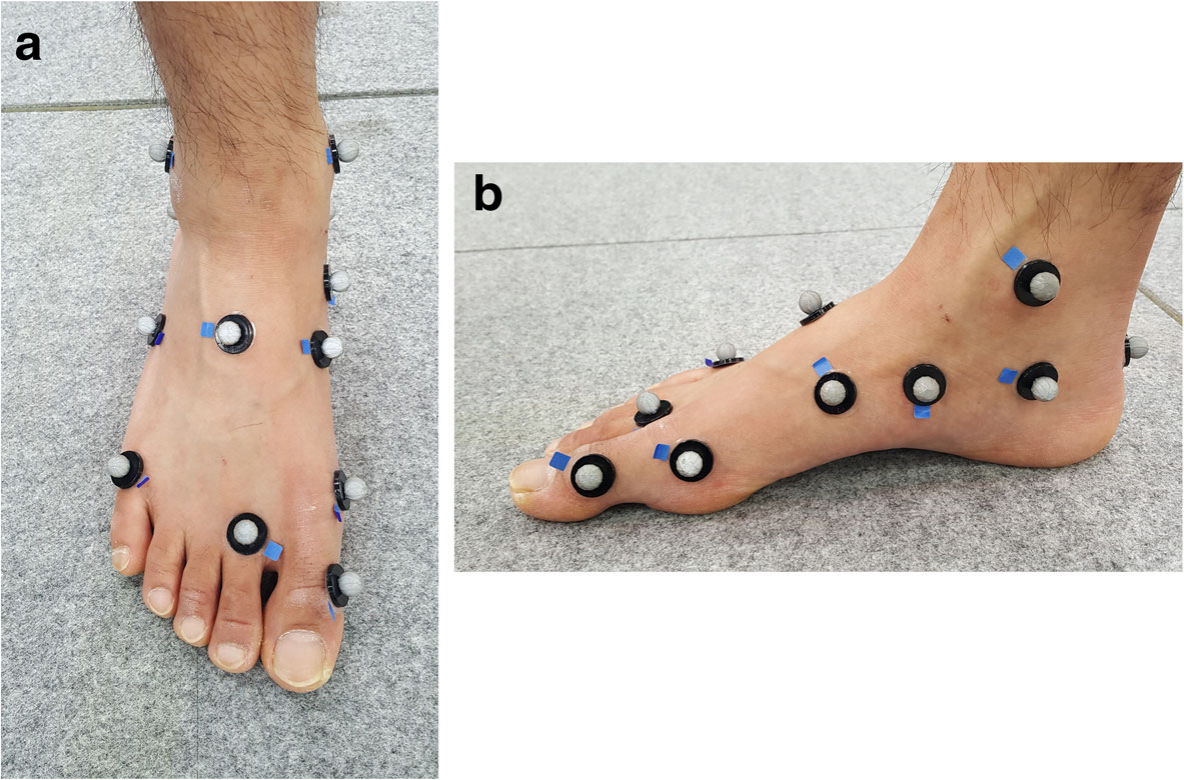
Anterior (**a**) and medial (**b**) views of the reflective marker position

The reflective markers (9.5 mm in diameter) were fixed to the right shank and foot at the most anterior prominence of the tibial tuberosity, most proximal apex of the fibula head, distal apex of the medial malleolus, distal apex of the lateral malleolus, Achilles tendon attachment, most medial apex of the sustentaculum tali, lateral apex of the peroneal tubercle, most medial apex of the tuberosity of the navicular, most lateral apex of the tuberosity of cuboid, dorso-medial aspect of the first metatarso-cuneiform joint (first metatarsal base), dorso-medial aspect of the first metatarso-phalangeal joint (first metatarsal head), dorso-medial aspect of the second metatarso-cuneiform joint (second metatarsal base), dorso-medial aspect of the second metatarso-phalangeal joint (second metatarsal head), dorso-medial aspect of the fifth metatarso-phalangeal joint (fifth metatarsal head), and most distal and dorsal point of the head of the proximal phalanx head of the hallux (Fig. 1a, b). The marker attachment was based on the foot model by Leardini et al. [22]. The repeatability [23] of this model has been confirmed in previous studies. Prior to data acquisition, static standing in the anatomical position was measured in each participant to calculate offset values for all joint rotation, which were eventually subtracted from the corresponding values over the walking stance.

The participants walked barefoot on a treadmill (Auto Runner AR-100; Minato Medical Science, Osaka, Japan) set to walking speed of 1.3 m s^−1^ because the coupling angle is very sensitive to small angle changes [3] and the difference in walking speeds among participants may increase variability of the coupling angle among participants. Walking speed in this study was set in reference to the speed used in previous studies [5, 24, 25], and this speed was verbally confirmed to each participant that it is roughly the usual speed preliminarily. During the tasks, measurements were performed by using a three-dimensional motion analysis system (Vicon Motion Systems, Oxford, UK) that included 13 infrared cameras. Before data collection, the participants were allowed to accustom themselves to the speed of the treadmill for at least 1 min. The walking biomechanics were continuously measured at 10 strides on the treadmill for each participant.

### Data analysis

Raw marker trajectory data were captured and filtered during walking, by using a second-order, zero-lag Butterworth, low-pass filter with a cutoff frequency of 6 Hz. The following four segments were defined in the kinematics model: (i) the shank comprising the tibia and fibula; (ii) the rearfoot (i.e., calcaneus); (iii) the midfoot comprising the navicular, cuneiform, and cuboid; and (iv) the forefoot comprising the first to fifth metatarsals. In this study, the three-dimensional joint angles were calculated at the distal segment, expressed relative to the adjacent proximal segment by using a right-handed orthogonal Cardan Xyz sequence of rotations (a sequence of plantarflexion/dorsiflexion, eversion/inversion, and abduction/adduction), which was selected to be equivalent to the joint coordinate system [24, 26, 27]. Hence, the joint angles were calculated as plantarflexion/dorsiflexion, eversion/inversion, and abduction/adduction of the rearfoot with respect to the shank, midfoot with respect to the rearfoot, and forefoot with respect to the midfoot.

The shank coordinate system was defined as described by Cappozzo et al. [28]. Briefly, the origin is located at the midpoint of the medial and lateral malleolus. The vertical axis (z) was defined as the projection of the line joining the origin and the tibial tuberosity on the frontal plane passing through the origin and the lateral malleolus and fibular head. The transverse axis (x) was orthogonal to the z-axis and lies in this frontal plane. The y-axis is orthogonal to the x and z planes.

The analysis interval in this study was only during the stance phase, same as the previous study [13]. Following calculation of the joint angles during the task, the data were time normalized to the stance phase (100 data points) at each stride.

### Calculation of the coupling angle

**Fig. 2:**
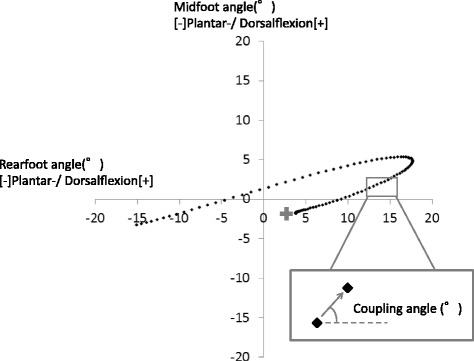
Angle-angle plot of rearfoot and midfoot. Coupling angle determined by the vector coding orientation between two adjacent data points in time on an angle-angle diagram. In this study, the vertical and horizontal axes were defined as distal and proximal segments, respectively. The plus (+) shows heel contact during walking. PF: plantarflexion, DF: dorsiflexion

Intersegmental coordination was inferred from a coupling angle (*γ*) (Fig. 2). The coupling angle was calculated by using the modified vector coding technique in this study [13] [Eq. (1)].

1 $$ {\gamma}_{j{,}_i}={\mathrm{tan}}^{-1}\;\left(\frac{y_{j,i+1}-{y}_{j,i}}{x_{j,i+1}-{x}_{j,i}}\right) $$

where 0^°^ ≤ *γ* ≤ 360^°^, *x*_*i*_, and *y*_*i*_ represent the proximal and distal joint angles, respectively. In addition, *i* represents the percent stance of the *j* th stride. To determine the coupling angle within a participant (i.e., ten strides) and among the participants, the mean coupling angle ($\overline{\gamma_i}$) was calculated from the mean *x*_*i*_ ($\overline{x_i}$) and the mean *y*_*i*_ ($\overline{y_i}$) at each percentage of stance [Eqs. (2), (3), (4), (5)]. The calculations were performed using the circular statistics [13].

2 $$ \overline{x_i}=\frac{1}{n}\;\sum_{j=1}^n\left(\cos\;{\gamma}_{j,i}\right) $$

3 $$ \overline{y_i}=\frac{1}{n}\;\sum_{j=1}^n\left(\sin\;{\gamma}_{j,i}\right) $$

4 $$ \overline{\gamma_i}={\mathrm{tan}}^{-1}\left(\overline{y_i}/\overline{x_i}\right)\kern0.5em \overline{x_i}>0 $$

5 $$ \overline{\gamma_i}=180+{\mathrm{tan}}^{-1}\left(\overline{y_i}/\overline{x_i}\right)\kern0.5em \overline{x_i}<0 $$

The length of the coupling angle ($\overline{R_i}$) was calculated according to Eq. (6). Finally, coupling angle variability (*CAV*_*i*_) was calculated among the participants using Eq. (7) [29].

6 $$ {\overline{R}}_i=\sqrt{{\overline{x_i}}^2+{\overline{y_i}}^2} $$

7 $$ {CAV}_i=\sqrt{2\cdot \left(1-\overline{R_i}\right)}\cdot \frac{180}{\pi } $$

### Categorization of coordination patterns

Intersegmental coordination was assessed as follows: between the rearfoot and midfoot plantarflexion/dorsiflexion, between the rearfoot and midfoot eversion/inversion, between the rearfoot and midfoot abduction/adduction, between the midfoot and forefoot plantarflexion/dorsiflexion, between the midfoot and forefoot eversion/inversion, and between the midfoot and forefoot abduction/adduction. The coupling angle represents an instantaneous spatial relationship from which four unique coordination patterns can be identified: (i) in-phase with proximal dominancy (the same direction and greater angular amplitude of proximal segment), (ii) in-phase with distal dominancy (the same direction and greater angular amplitude of distal segment), (iii) anti-phase with proximal dominancy (the opposite direction and greater angular amplitude of proximal segment), and (iv) anti-phase with distal dominancy (the opposite direction and greater angular amplitude of distal segment) [18]. For example, the in-phase with proximal dominancy in the rearfoot and midfoot coordination is adjoining segments that rotate in the same direction and greater angular amplitude of the rearfoot compared to midfoot motion. In the present study, the positive direction (+) of segmental rotation was defined as dorsiflexion, inversion, and adduction.

**Fig. 3:**
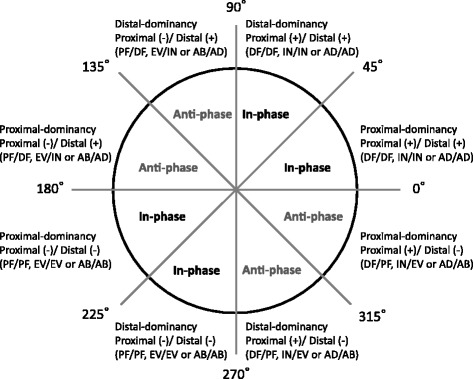
New coordination pattern classification. The coupling angle was categorized into four coordination patterns as follows: in-phase with proximal dominancy, in-phase with distal dominancy, anti-phase with distal dominancy, and anti-phase with proximal dominancy. The positive direction (+) represents dorsiflexion (DF), inversion (IN), and adduction (AD). The negative direction (−) represents plantarflexion (PF), eversion (EV), and abduction (AB)

The stance phase was divided into the early stance (1%–33%), midstance (34%–66%), and late stance (67%–99%) based on a previous study [13]. These phases represent the loading response, midstance, and propulsion, respectively. Finally, the mean coupling angles were categorized into one of the four coordination patterns at each phase (Fig. 3; the unit circle was divided into 45° bins).

## Results

### Coordination patterns between the rearfoot and midfoot segments

**Fig. 4:**
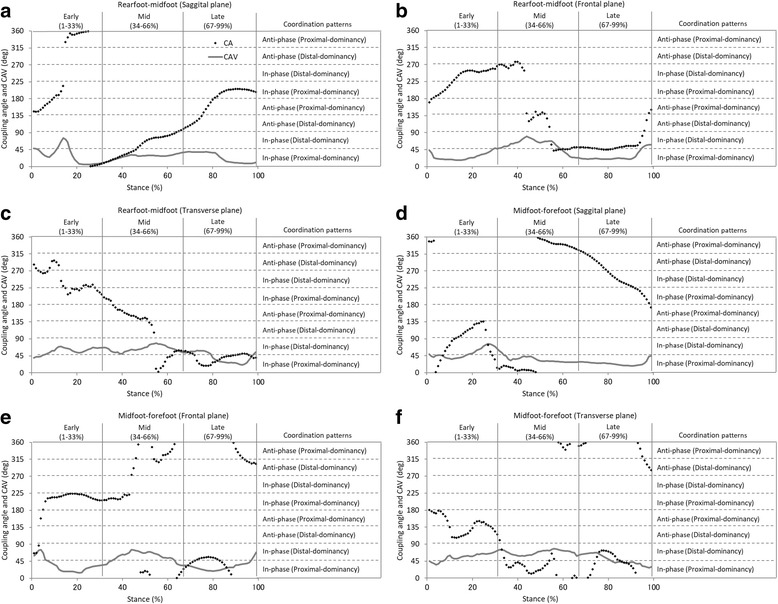
Mean coupling angle and coupling angle variability for inter-segment coordination during walking (**a**, **b**, **c**, **d**, **e** and **f**). Stance is divided into early, mid, and late

**Fig. 5:**
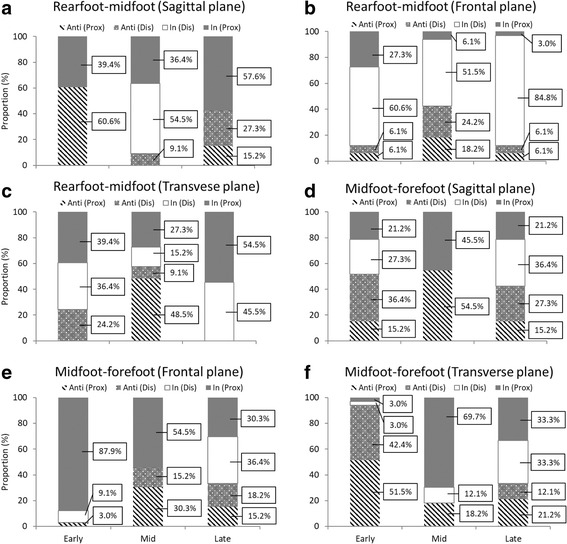
Stacked graph for inter-segment coordination during walking and coordination pattern (**a**, **b**, **c**, **d**, **e** and **f**). In: in-phase, Anti: anti-phase, Prox: proximal dominancy, Dis: distal dominancy

The mean coupling angles for-intersegmental coordination and proportion of coordination pattern are shown in Figs. 4 and 5, respectively. In the sagittal plane, the rearfoot-midfoot coordination pattern started from the anti-phase with proximal dominancy and rapidly changed to in-phase with proximal dominancy. The coupling angle variability (CAV) showed peak value at approximately 10% in early stance. The coordination pattern moderately changed in the midstance and late stance (Fig. 4a). In the frontal plane, the rearfoot-midfoot coordination pattern in early stance was observed to be mostly in-phase with the proximal and distal phases. The coordination pattern rapidly changed at approximately 45% of the midstance, and the CAV showed peak value in this phase. In the late stance, the coordination patterns were mostly in-phase with distal dominancy and changed to anti-phase with proximal dominancy before the push-off (Fig. 4b). In particular, the coordination pattern with the highest proportion was in-phase with distal dominancy (the midfoot eversion is greater than the rearfoot eversion) in the early stance (Fig. 5b). In the transverse plane, whereas the rearfoot-midfoot coordination pattern considerably changed in the early stance and the midstance, it was maintained after in-phase with proximal and distal phases in the late stance. Overall, the CAV showed a relatively high value in the transverse plane (Fig. 4c).

### Coordination patterns between the midfoot and forefoot segments

Concerning the midfoot-forefoot coordination pattern in the sagittal plane, although it changed from in-phase with proximal dominancy to anti-phase with distal dominancy in the early stance, it transitioned back to in-phase with proximal dominancy at midstance. The CAV showed peak value at the end of the early stance (Fig. 4d). In the frontal plane, the midfoot-forefoot coordination pattern started from in-phase with distal dominancy, and then rapidly changed. Subsequently, the midfoot-forefoot coordination pattern remained as in-phase with proximal dominancy. In the midstance and late stance, the midfoot-forefoot coordination showed various patterns. The CAV was observed to have a high value after the initial contact and at approximately 45% of the midstance (Fig. 4e). In particular, the coordination pattern with the highest proportion was in-phase with proximal dominancy (the midfoot eversion is greater than the forefoot eversion) in the early stance (Fig. 5e). In the transverse plane, the midfoot-forefoot coordination pattern considerably changed throughout the stance, whereas the CAV showed a high value throughout the whole stance (Fig. 4f).

## Discussion

Understanding the coordination patterns between segments is essential in elucidating the mechanism for the occurrence of injuries [10] because a disruption in the inter-segmental coordination may predispose injuries [30]. However, because the traditional ratio method calculates a singular value for the whole stance, the interaction between segments with and without injuries cannot be detected. A recent study [10] investigated the interaction between foot pronation and tibial internal rotation with and without anterior knee pain by using the ratio and vector coding techniques, and the results showed a significant difference in the coupling pattern only when the evaluation was performed with the vector coding technique. Chang et al. [13] modified the vector coding technique and classified the four coordination patterns (in-phase, anti-phase, proximal-phase, and distal-phase); further, a recent study [16] categorized the coordination of the intersegmental foot kinematics based on the method of Chang et al. [13]. However, the in-phase or anti-phase categories in this method only provide adjoining segments rotating in the same or opposite direction. That is, the in-phase and anti-phase provide no information on which segment is dominant regarding the angular amplitude in the proximal or distal segments. To investigate the factors of the injury occurrence, previous studies [7, 8, 31] have used the ratio of the angular peak value of two segments. For example, Williams et al. [8] reported that low-arched individuals with higher rearfoot eversion/shank internal rotation ratio (i.e., relatively more rearfoot motion) had a higher incidence of knee-related injuries. That is, because the difference in the relatively angular amplitude of two segments is related to the occurrence of injuries, understanding the dominant angular amplitude is important. In the present study, the dominancy of angular amplitude in the proximal or distal segments can be determined in all coordination patterns. The collected data would help in ensuring a better understanding of the interaction of the intersegmental foot kinematic time series, and may better facilitate the interpretation of coordination patterns.

Furthermore, the present study is the first to quantify the coordination among the rearfoot, midfoot, and forefoot during walking using a coordination pattern classification [17]. First, the present study showed results similar to those of an in vivo measurement of the rearfoot, midfoot and forefoot during walking [32]. Buldt et al. [2] showed the range of motion of the rearfoot (9.2 degree) in the frontal plane during walking in a normal foot is greater than that of the midfoot (6.0 degree). However, this method cannot adequately evaluate evaluate a range of motion (i.e. angular amplitude) in a time series. While previous studies [13, 17] have investigated intra-foot coordination during walking, it is different from the present study because midfoot segment was not included. To our knowledge, only Arnold et al. [16] have quantified the coordination among the rearfoot, midfoot, and forefoot during walking, which has shown that coordination between the rearfoot and midfoot was partly in phase during the early phase with young adults. This result was partially consistent with the findings of this study. However, this information being in phase does not show which of the angular amplitudes of the rearfoot or the midfoot substantially move. Conversely, the results in the present study demonstrated the rearfoot-midfoot coordination during the early stance was mainly in phase with distal dominancy. Further, the midfoot-forefoot coordination was mainly in phase with proximal dominancy. Hence, this study provided information on both in phase and dominancy of the angular amplitude; the results showed that the midfoot, rather than the rearfoot and forefoot, dominantly everts.

The results obtained in the current study may be used in the future for comparison with data on foot deformities, running injuries, or the elderly population. A previous study [29] suggested that the coordination patterns can be adversely influenced by pathology. Rodrigues et al. [10] showed that runners with anterior knee pain have different coupling angles between the internal rotation of the shank and eversion of the rearfoot compared to those without anterior knee pain, which suggests that this alternation is useful for the detection of injuries. A recent study [2] has reported that the range of motion of the midfoot in the transverse plane during walking in patients with pes planus was significantly narrower than that in patients with pes cavus. Thus, in patients with pes planus, the coupling angles and coordination patterns in the transverse plane may differ from the results obtained from healthy participants in the present study. Hence, the results of this study could be used as data to distinguish the presence of injuries or abnormal inter-segmental foot motions such as pes planus. Additionally, Arnold et al. [16] showed that the coordination between the rearfoot and midfoot in the frontal plane during the early stance was more frequently in phase in older adults compared to younger adults; they have implicated that older adults are adaptive in their stability by reducing intrinsic foot mobility. Thus, this study will provide comparable data to evaluate the stability of the foot. The results in this study may also be helpful in considering clinical assessment and treatment interventions.

The present study has certain limitations. First, one major limitation of the present study pertains to the use of skin markers to track the underlying skeletal structure. While the foot model used in this study has been confirmed by validating an in vitro study [33], the validation of this model has not been investigated for an in vivo study. Thus, skin markers mounted on externally identifiable bony landmarks in the foot may not follow for the underlying individual skeletal segments to be properly evaluated during running. Secondly, the participants’ foot posture during standing was only visually inspected to eliminate obvious pes planus. Because the result of the visual assessment is qualitative, it may not strictly distinguish a normal foot from one with pes planus. Therefore, foot posture may need to be assessed using a quantitative method such as the foot posture index [34]. Finally, the walking speed in this study was set to 1.3 m s^−1^. While the speed of 1.3 m s^−1^ was verbally confirmed with each participant that it is roughly equivalent to the usual walking speed, it may be different from the real usual walking speed. Also, because such standardized spatial-temporal parameters may have affected results in this study, the walking speed may need to be set as a comfortable speed for each individual participant. These limitations will need further investigation in the future.

## Conclusion

The present study investigated the coordination among intersegmental foot kinematics during walking. The notable point in this study is to quantify the coordination of intersegmental foot kinematics during walking by using a new coordination pattern classification. The present study elucidated that the midfoot, rather than the rearfoot and forefoot, dominantly everts in the early stance. The results of the present study could help to clarify the interaction of intersegmental foot kinematic time series during walking. The coordination patterns can also be adversely influenced by pathology [3, 28], and the change of intersegmental coordination is related to injuries [10]. Thus, the results of this study could be used as data to distinguish the presence of injuries or abnormal inter-segmental foot motions, such as pes planus. Furthermore, these data might be used in the future for comparison with data on foot deformities, running injuries, or with those from the elderly population.

## Abbreviation

CAV: Coupling angle variability
